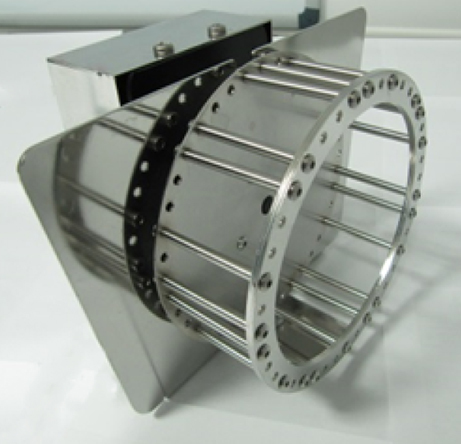# Measuring motor deficits in mice

**Published:** 2014-03

Many human neurological, neuromuscular and musculoskeletal diseases involve deficits in motor function. Currently, investigations into disease onset and progression in mouse models of these conditions depend on somewhat unreliable tests that only measure a few motor functions. Now, Mandillo et al. validate an automated home-cage wheel-running system for the measurement of early motor deficits in mouse disease models. By undertaking studies at two centres, the researchers show that the system consistently detects differences in performance among four inbred strains of mice. They also report that the system allows the detection of very early motor function deficits in mutant mouse models of two progressive neurodegenerative diseases and in a mouse strain with subtle gait abnormalities. This new test therefore provides a reliable method for detecting motor deficits at pre-symptomatic stages in mouse disease models that should be useful in longitudinal investigations of potential therapeutic agents. **Page 397**

**Figure:**